# A Modified Aquila-Based Optimized XGBoost Framework for Detecting Probable Seizure Status in Neonates

**DOI:** 10.3390/s23167037

**Published:** 2023-08-09

**Authors:** Khondoker Mirazul Mumenin, Prapti Biswas, Md. Al-Masrur Khan, Ali Saleh Alammary, Abdullah-Al Nahid

**Affiliations:** 1 Electronics and Communication Engineering (ECE) Discipline, Khulna University (KU), Khulna 9208, Bangladesh; k.mirazulmumenin@gmail.com (K.M.M.); pbs86865@gmail.com (P.B.); 2 Department of ICT Integrated Ocean Smart Cities Engineering, Dong-A University, Busan 49315, Republic of Korea; almasrurkhan@donga.ac.kr; 3 College of Computing and Informatics, Saudi Electronic University, Riyadh 11673, Saudi Arabia

**Keywords:** neonatal seizure, EEG, shallow learning, classification, optimization

## Abstract

Electroencephalography (EEG) is increasingly being used in pediatric neurology and provides opportunities to diagnose various brain illnesses more accurately and precisely. It is thought to be one of the most effective tools for identifying newborn seizures, especially in Neonatal Intensive Care Units (NICUs). However, EEG interpretation is time-consuming and requires specialists with extensive training. It can be challenging and time-consuming to distinguish between seizures since they might have a wide range of clinical characteristics and etiologies. Technological advancements such as the Machine Learning (ML) approach for the rapid and automated diagnosis of newborn seizures have increased in recent years. This work proposes a novel optimized ML framework to eradicate the constraints of conventional seizure detection techniques. Moreover, we modified a novel meta-heuristic optimization algorithm (MHOA), named Aquila Optimization (AO), to develop an optimized model to make our proposed framework more efficient and robust. To conduct a comparison-based study, we also examined the performance of our optimized model with that of other classifiers, including the Decision Tree (DT), Random Forest (RF), and Gradient Boosting Classifier (GBC). This framework was validated on a public dataset of Helsinki University Hospital, where EEG signals were collected from 79 neonates. Our proposed model acquired encouraging results showing a 93.38% Accuracy Score, 93.9% Area Under the Curve (AUC), 92.72% F1 score, 65.17% Kappa, 93.38% sensitivity, and 77.52% specificity. Thus, it outperforms most of the present shallow ML architectures by showing improvements in accuracy and AUC scores. We believe that these results indicate a major advance in the detection of newborn seizures, which will benefit the medical community by increasing the reliability of the detection process.

## 1. Introduction

Neonatal seizure is a common neurological condition that affects the brains of newborns, both preterm and term. It is characterized by abnormal electrical signals in the brain, which negatively impact neurodevelopmental outcomes. The incidence of clinically evident neonatal seizures is estimated to be three in one thousand for full-term neonates and fifty in one thousand for very preterm babies [1]. Among preterm and full-term newborns, preterm neonates are particularly at risk for seizures, with a higher incidence occurring during the first week after birth. Though the exact number of incidents is unknown, the approximate number can be identified according to the geographical settings. Table 1 represents the incidents of neonatal seizures in the USA, Canada, the UK, and India.

Seizures in neonates can range from mild to severe and cause symptoms such as sudden unusual shaking, abnormal body movements, and excessive eye blinking. The underlying causes of these seizures are diverse and may include intracranial hemorrhage (ICH) [6], urinary tract infections (UTI) [7], hypoxic ischemic encephalopathy (HIE) [8], etc. These seizures can lead to long-term consequences such as epilepsy, developmental delay, anxiety attacks, and other neurological disorders. Early detection of neonatal seizures is crucial, as it allows for prompt medical intervention and can reduce the damage caused by the seizure. Additionally, timely seizure detection can greatly aid in controlling the effects of seizures and improve the patient’s condition. However, diagnosing seizures in neonates can be challenging, as the seizure time is often short and indistinct. The immature brains of neonates often produce unique electrical signals that are difficult to differentiate from normal signals, and seizures in neonates can go undetected as they may not show observable changes in brain signals. While several tests and methods are available for detecting neonatal seizures, electroencephalography (EEG) is considered the most reliable and efficient option. EEG is a powerful tool that is capable of detecting brain signals, encompassing multiple types of brain waves: beta, alpha, theta, hi-beta, gamma, and delta. Additionally, the non-invasive nature of EEG makes it a simple and convenient method for mapping brain networks without the need for surgery. Neurologists can detect seizures by EEG through the mapping, as abnormal electrical signals of the brain are shown in cases of seizures. Although this is an accurate brain mapping process, EEG evaluation requires highly skilled medical personnel as well as various specialized equipment. This evaluation process is time-consuming, exhausting, and requires consistent expertise. Neurologists may overlook minor changes in brain waves detected by EEG and conclude that there was no seizure, even if the brain signals were abnormal.

In underdeveloped and developing countries, the lack of neurologists often becomes an alarming issue as the patients have to wait for a long time to obtain seizure detection results. If the detection procedure takes a long time, the incidence of seizures can increase. Consequently, to omit the limitations of current clinical practices for neonatal seizures, it is crucial to explore alternative methods that are more effective and efficient. Recent literature shows that the adoption of new techniques for accurate and effective seizure detection has recently increased due to technological improvements. These new techniques are incorporated into ML algorithms to distinguish between seizure patients and normal patients. Furthermore, it can alleviate the tedious work associated with traditional EEG examination processes. By leveraging the expertise of ML experts in collaboration with neurologists, this approach has the potential to bring about revolutionary change and address various biomedical challenges related to this field. Several studies have used ML algorithms to discriminate between seizure and non-seizure states, but none of them have applied hyper-parameter optimization using meta-heuristic optimization algorithms to improve their performance. So, considering this research gap, in this paper, we incorporated a meta-heuristic optimization algorithm with present ML models to make an effective and accurate framework for detecting the seizure condition. The primary contributions of this research study can be listed as follows:

- Develop an ML framework for reasonable seizure detection;
- Modify the Aquila Optimization (AO) algorithm for better convergence with respect to epochs, having lower latency;
- Optimize hyper-parameters for better performance using a modified MHOA.

The overview of the rest of this research is as follows: The existing neonatal seizure detection techniques were discussed briefly in Section 2, Section 3 provides insight into the methodology used in this research, Section 4, illustrates and compares our findings with relevant state-of-the-art research, and finally, Section 5 draws the conclusions and provides scope for future research.

## 2. Literature Review

There have been several studies regarding the use of ML and DL in the neonatal seizure detection process. These studies have used various classifiers to provide better outcomes when detecting neonatal seizures. R. Elakkiya [9] proposed an ML approach for detecting neonatal epileptic seizures using three robust algorithms: SVM, ANN, and 1D-CNN. The achieved accuracies were 92.30%, 88%, and 95.99%, respectively. Tapani et al. [10] created a support vector machine (SVM) model to identify the non-stationary periodic attributes of neonatal seizures. The algorithms were applied to multi-channel recordings and demonstrated an AUC of 98%. No hardware implementation was used, which reduced the clinical feasibility of these two studies. Nagarajan et al. [11] proposed an ML architecture operating with comparable predictive performance with a minimum level of configuration. The classification model consists of a scalable and binary classifier called ProtoNN. It is a K-Nearest Neighbors Algorithm (KNN) that can handle the tradeoff between the model size and prediction accuracy. The architecture proposed in this research has a sensitivity of 87%, which is higher than that of related previous research. The CNN model has been proven to be a robust tool for extracting the necessary features. A new seizure detection algorithm was proposed by Seungjun Ryu et al. [12], which uses the principal component analysis (PCA) to extract features and compare them with other ML algorithms. Four prediction models were used, which included LR (logistic regression), dense trees, 2D-SVM (support vector machine), and cos-KNN (cosine k-nearest neighbor). As the data dimensions were reduced by using the PCA, the performance of training and test data improved. Karoliina T. Tapani [13] proposed an SVM-based SDA using a different data set of 28 neonates. The performance of the initial training set was cross-validated against the performance of the validation set to assess its generalizability.

Khadijeh Raeisi et al. [14] presented a DL-based Graph Convolutional Neural Network (GCNN) for automatic seizure detection. Their findings demonstrate that functional connectivity measures derived from EEG graph representations can effectively take advantage of the dependencies between EEG data and result in the accurate diagnosis of newborn seizures. Amr Zeedan et al. [15] implemented automatic DL models: two models were based on feed-forward neural networks, and one model was based on LSTM. The accuracy levels of these models were 74.3% and 74.3%, respectively. Frassineti et al. [16] implemented such a hybrid system, which is a combination of deep neural networks and stationary wavelet transforms (SWT). The use of SWT increased the robustness of the proposed methods by 5% compared to the process where a raw EEG time series was used. This research is one of the first successful approaches using hybrid techniques. L. Webb et al. [17] proposed a residual deep neural network that was trained using 10-fold cross-validation. It demonstrated the highest accuracy level of (95%), with a median accuracy of 91% for each patient. A newly added DL method was used in the research of M. Asjid Tanveer et al. [18], which is used for both neonatal seizure detection and classification. They made a two-dimensional convolutional neural network (CNN) that classifies the seizure and non-seizure states from the raw data. Three separate 2D CNN models were trained on the dataset, and the average accuracy levels were 95.6%, 94.8%, and 90.1%, respectively. Caliskan et al. [19] proposed a transfer learning technique for reconstructing some pre-trained deep convolutional neural networks (p-DCNN) for neonatal seizure detection. Gramachi et al. [20] developed a sliding window design for the training data generation process. This design increases the amount of data available to feed into neural networks on a large scale. A CNN model was also developed to train the dataset and detect seizure episodes, which showed an accuracy level of 96% to 97%. This study helped the researchers to analyze their dataset by performing some modifications.

DL and ML algorithms were used in the majority of the studies for neonatal seizure classification using EEG signals. However, the use of DL-based methods for this purpose can create several complexities that can affect the proper implementation of the methods. Additionally, it requires a large dataset to apply DL models that might not work well for smaller datasets. In this study, several ML models were used for neonatal seizure classification. Fifteen features were extracted from the dataset, and several ML algorithms such as RF, DT, GBC and XGboost were applied to gain the best prediction result. Table 2 portrays a list of papers related to our study, where the details of the publisher, trained algorithm, used network, and final result of each paper are included.

## 3. Materials and Methods

In this study, we aimed to address the challenges of detecting neonatal seizures through a binary classification problem. We utilized a dataset from the Neonatal Intensive Care Unit (NICU) of Helsinki University Central Hospital, Finland, which consists of data from 79 neonates and was collected using the international 10–20 EEG system [22]. To make these raw data usable for our classification models, we applied various preprocessing techniques and a few feature extraction methods.

**Figure 1:**
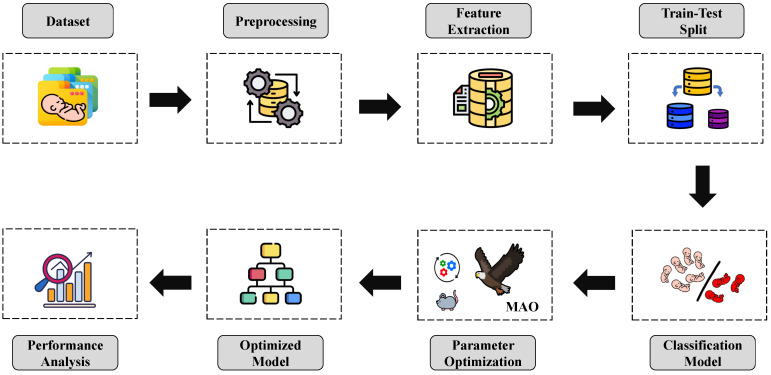
Workflow diagram of the proposed system.

To classify the data, we have utilized DT, RF, GNB, and XGBoost as classifiers. The performance of the models has been evaluated with a few standard performance evaluation metrics. The results of these evaluations are discussed in the next section. Figure 1 demonstrates a visual representation of the workflow of this paper.

### 3.1. Dataset

This dataset contains 21–channel EEG measurements conducted at the NICU (newborn intensive care unit) of Children’s Hospital, Helsinki University Central Hospital, Finland, and a total of 79 neonates participated [22]. To train and test the algorithm for EEG signal classification, these data were sampled with a NicOne EEG amplifier, where the sampling frequency was 256 Hz. The median recording duration of the multi-channel EEG was 74 min, and each recording duration was approximately 1 h. The experts annotated seizures as abnormal electrographic events, where sharp and rhythmic waveforms are seen in the beginning and end. If the duration of this state remains for more than 10 s, it is classified as a seizure. Among the three experts working with this dataset, each expert identified 460 seizures and agreed that 22 neonates were free from any seizure attack. On the other hand, seizures were detected in 39 neonates. All of these recorded data are available as EDF files. The annotations are stored in both .mat file format and CSV file format. Each .mat file has a cell array of 79 elements. For each element, there is an (M,N) array, where M describes the number of experts and N describes the annotation duration (seconds). Three CSV files (A, B, and C) are used for storing the annotations. Each CSV file contains the annotations of an expert, of which there are 79 columns. In the CSV files, each column represents a study ID number, and each row represents one second of EEG recording where 1 defines seizure and 0 defines a non-seizure. Measurements of EEG spectral power were used to summarize the EEG recordings, and the noise floor was measured using the power spectral density. Public distribution of these files is permitted by the local ethics committee of the Children’s Hospital, Helsinki University Hospital, Finland. Three files were discarded after extraction, as they were found to be corrupted. In this dataset, more seizures were detected with high efficiency because of the use of 10–20 EEG systems with 19 electrodes. So, this dataset could be a huge opportunity for comparing and finding the relations and interactions between seizure location and EEG montages.

### 3.2. Data Preparation

The initial stage in every ML pipeline is to clean up the messy raw data and convert them into a form in such a way that they can be used by the classification models. Real-world data can be extremely large, have numerous errors such as missing values, and have irrelevant feature variables, making it inappropriate for use in a few circumstances. So, we prepared the raw data before training the models so that a higher accuracy could be acquired, and the processed data could be more accessible for the users. For our dataset, extensive preprocessing was carried out, which is discussed in the next section.

#### Data Preprocessing

The raw EEG data were visualized using EEGLAB, and it was found to contain 21 channels. Among them, few showed characteristics of ECG signals, so that were discarded, and a total of 18 channels remained. At first, raw EEG data were downsampled from 256 HZ to 32 Hz for convenience [23,24].

**Figure 2:**
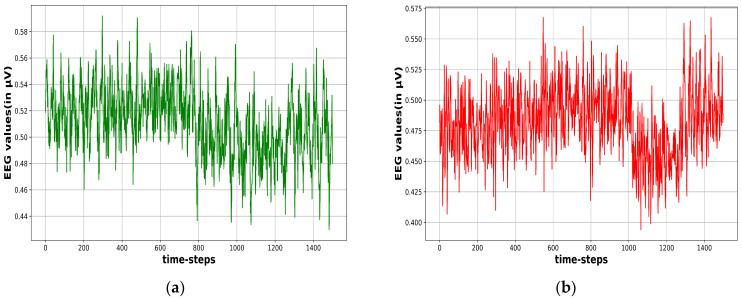
(**a**) EEG activity in non-seizure situations and (**b**) EEG activity in seizure situations.

**Figure 3:**
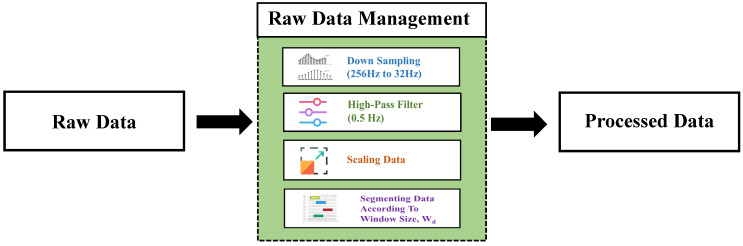
Data preprocessing steps.

A high-pass filter is typically used to remove low frequencies below 0.1 Hz, and occasionally even 1 Hz from these signals. In addition, a low-pass filter was used to filter out frequencies greater than 40 to 50 Hz [25]. For our approach, the data were filtered using a high-pass filter with a cut-off frequency of 0.5 Hz. Filtered data were then re-scaled to values between 0 to 1 by applying a min–max scaling method. The data from each channel were then divided into eight-second windows and preserved as one-dimensional windows for each window length [11]. For the annotations, we compared the number of seizure annotations in separate windows and transformed the multi-channel output annotations to either 0 (indicating non-seizure) or 1 (for seizure). A window was labeled as 1 if it surpassed a predefined threshold value (VT). Figure 2 contrasts the EEG signal activity under ictal and non-ictal conditions. Signal activity is seen in Figure 2a under normal circumstances. Increased signal activity is shown in Figure 2b when a seizure occurs. The following procedures were used to construct the dataset, and Figure 3 shows a visual depiction of the data preparation procedure.

### 3.3. Feature Extraction

A feature is a distinguishing quality, an identifiable measurement, and a functional component obtained from a portion of a pattern [26]. Features contribute to the ML algorithm to solve specific tasks. In the field of seizure detection, a robust feature extraction approach is necessary.Each EEG window segment was converted into 15 human-engineered features in this study, based on their consistency in prior research [10]. Among these 15 features, 11 of them were time domain features, and the rest of them were entropy domain features. The features used were simple EEG signal features, including statistical features such as the mean, median, variance, standard deviation, skewness, kurtosis, and similar [27], as presented in Table 3. The rest were entropy-based features that determine the uncertainties and complexities of decomposed signals.

### 3.4. Training/Test Set Split

In this work, we split the dataset into a ratio of 70:30 for the training set and test set. The training dataset was used to train our ML models, and the test dataset was used to validate our trained models.

### 3.5. Classification Model

Classifying seizure and non-seizure signals is a challenging task as errors can occur if the models are not configured properly. However, the probability of the system performance might increase if a classification model is properly tuned. A classification model will attempt to draw a certain conclusion from the input signals during training and predict the class, i.e., whether it is seizure or non-seizure, for the new data. There are a few ML classification models available, and DT, RF, GBC, and XGBoost were used in this study.

#### 3.5.1. DT

A non-parametric supervised learning technique called DT is used for both classification and regression problems. It is distinguished by a hierarchical tree structure made up of a root node, internal nodes, branches, and leaf nodes [28]. To find the root node and decision node of a decision tree, a metric is used called ‘entropy’ to measure the uncertainty in a dataset. It can be mathematically shown,

(1) $$Entropy\left(s\right)=-proba_{\left(+\right)}〖log_{p}{〗}_{\left(+\right)}-proba_{\left(-\right)}〖log_{p}{〗}_{\left(-\right)}$$

where *s* is the subset of the training sample, $proba_{\left(+\right)}$ is the probability of a positive class, and $proba_{\left(-\right)}$ is the probability of a negative class.

There are two types of DT: categorical variables and continuous variables [29]. In the categorical DT, every stage of the decision process can fall into either one of the categories: yes or no. On the other hand, the continuous variable DT predicts results based on available information and parameters.

#### 3.5.2. RF

RF is a classification algorithm that is a combination of several individual DTs. It represents a collection of results from different trees [30]. In the Random Forest, the Gini (G) index is frequently used to decide how nodes are arranged on decision tree branches when working with categorical data. The formula is

(2) $$G=1-\sum_{i=1}^{c}{\left(p_{i}\right)}^{2}$$

where $〖(p_{i})〗$ is the relative frequency of the class that is observed in the dataset and *C* is the number of classes.

To identify which branch on a node is more likely to occur, this formula calculates the Gini for each branch using the class and probability.

#### 3.5.3. GBC

The Gradient Boosting Classifier (GBC) is a bunch of ML techniques that combine various weak learning models to produce a robust predictive model; this framework permits the use of any differentiable loss function [31]. As a result, it is not necessary to create brand new boosting algorithms for every desired loss function.The fundamental elements of gradient boosting consist of a feeble predictor for forecasting, a loss function for optimization, and an incremental model for progressively incorporating feeble predictors. This can be formulated as

(3) $$The\;combined\;prediction\;F_{1}=F_{0}+\theta \times Y_{1}$$

where $F_{0}$ is the initial prediction, θ is the learning rate, and $Y_{1}$ is the first tree prediction.

The GBC has a tendency to overfit a training dataset rapidly [32]. However, regularization techniques can be employed to control various aspects of the algorithm and generally improve its performance by reducing overfitting.

#### 3.5.4. XGBoost

XGBoost is used for supervised learning problems. It is an ML algorithm and provides a highly accurate implementation of a gradient-boosting framework [33]. It combines a set of weaker models to provide accurate predictions, such as

(4) $$Predicted\;value,〖y^{\prime }{〗}_{i}=\sum_{m=1}^{m}f_{k}$$

where *m* is the number of decision trees, $f_{k}$ is the prediction from a decision tree, and $〖y^{\prime }{〗}_{i}$ is the feature vector at $i_{th}$ data point.

There are several parameters of XGBoost that are optimized depending on the performance of the validation set. These parameters might improve the performance of the algorithm. While detecting seizures, a mismatch in the classification process affects the prediction accuracy. By using the XGBoost classifier, the mismatch in training and testing data in the classification process have been reduced for higher accuracy in the prediction models [34].

### 3.6. Parameter Optimization

Supervised learning plays a vital role in modern ML approaches. There are a few supervised ML classifier models available, and a few of them were invented at an early age. With the advantages of the mathematical model and optimization techniques, recently a few advanced ML classifier models have been introduced, such as DT, RF, GBC, and XGBoost.

In this study, we chose XGBoost as it has achieved state-of-the-art results on many ML challenges and has demonstrated excellent performance in solving various EEG-related classification problems [35,36]. XGBoost is also highly scalable and requires minimal resources for algorithmic optimization [37,38]. In addition, when it was used on our dataset, XGBoost was able to outperform all other classifiers, which is another indicator of the suitability of XGBoost for solving the classification problem at hand. Table 4 depicts a comparison of the performances of the different classifiers. Here, ACC, Re, F1, and K stand for the accuracy score, recall score, F1 score, and kappa score, respectively.

However, these outcomes fall short of what was expected. As a result, we fine-tuned the parameters of our proposed model using a meta-heuristic approach to increase its accuracy and resilience in identifying newborn seizures. When it comes to optimization, meta-heuristic algorithms outperform more conventional algorithms. Numerous categories of meta-heuristic optimization algorithms exist, such as Swarm Intelligence (SI), evolutionary, physics-based, and human-based algorithms. We prefer a population-based algorithm, named Aquila Optimization (AO).

### 3.7. Parameter Tuning with Modified Aquila Optimization (M-AO)

ML models generally contain a few parameters, which can be adjusted to obtain a reasonable performance. Hyper-parameter tuning is required to obtain a desirable performance with reasonable latency [39]. Numerous optimization techniques are available, including exhaustive search, gradient descent, and genetic algorithms, etc., [40]. In this study, we used the M-AO for hyper-parameter optimization of XGBoost. The range of the hyper-parameters in the XGBoost classifier is shown in Table 5.

Therefore, the whole process of hyper-parameter tuning is referred to as an optimization problem. In our proposed approach, a generalized framework to optimize the hyper-parameter of XGBoost can be formulated as follows:

(5) $$func_{obj}\left(XGB\left(P\right);P\right),$$

(6) $$p=h_{1},h_{2},h_{2},\ldots h_{n}$$

In Equation (6), h∈p refers to the hyper-parameters of the XGBoost classifier. In this study, we used accuracy as the objective function for the optimization task. The mathematical generalization can be illustrated in the equation

(7) $$func_{obj}=\;\;1-Accuracy\;Score\left(XGB\left(P\right)\right),$$

where the *Accuracy Score* of the XGBoost classifier is formulated as

(8) $$Accuracy\;Score=\frac{AS}{TS}\;.$$

In Equation (8), *AS* and *TS* refer to the number of accurately classified samples and the total number of samples, respectively. To perform neonatal seizure detection, a modified version of the Aquila Optimizer (AO) [41] was implemented to tune the hyper-parameters of the proposed framework. In the proposed modified AO-based ML framework for neonatal seizure detection, the phases of our framework are delineated sequentially, as follows.

#### 3.7.1. Proposed Model

According to [41], despite the robust performance of the AO, its suitability and efficacy may diminish in the face of escalating complexity in real-world problem domains, potentially leading to sub-optimal outcomes or convergence on local optima, as the second searching step of the AO utilizes the Levy Flight Distribution to control the search space of Aquila, which can limit search space of Aquila and may lead these solutions to fall in local optima. Furthermore, in the third searching step the two parameters that determine the exploitation phase are taken as constants, which can result in weak local exploitation. To further improve the performance and contaminate these drawbacks, Improved Aquila Optimization (IAO) was proposed by [42]. With the introduction of a function named the “Search Control Factor (SCF)”, which decreases as the iterations progress, this SCF eradicated the problem regarding the Levy Flight Distribution and expanded the search range area of Aquila. Additionally, the Random Opposition-Based Learning (ROBL) and Gaussian Mutation (GM) strategies were added to further improve the exploration and exploitation phase.

##### Modified Aquila Optimization

In this paper, we introduced a modified version of Aquila Optimization (M-AO). We were inspired to make further changes to AO by adjusting the SCF from IAO. However, in IAO, we found that the convergence property of SCF slows down the performance with the epochs. This convergence property might provide a few challenges to find an optimal solution. To address this issue, we propose a modified version of IAO, which incorporates a Modified Search Control Factor (M-SCF) that is specifically tailored to the second and third searching steps. The following sections provide a comprehensive overview of the M-AO approach, highlighting the specific modifications that have been made and their impacts on the optimization process.

##### Explanation

M-SCF controls the search range of Aquila, decreasing Aquila’s movement faster with respect to the epoch. Consequently, the search range will be much narrower compared to the previous SCF. Moreover, the optimal solution can be found much more quickly than in the previous method. The modified M-SCF can be represented as

(9) $$M-SCF\left(t\right)=2\times exp\;\left(1-\left(\frac{t\times \left(t\times 0.1\right)}{T}\right)\right)\;\times \;dir.$$

(10) $$dir=\left\{\begin{array}{cc} 1 & if\;r<0.5,\\ -1 & else. \end{array}\right.$$

In this equation, *t* is the current iteration and *T* is the maximum iteration. The variable *r* represents a random number ranging from 0 to 1, while dir is the direction control factor, as defined in Equation (10). This factor plays a crucial role in controlling the flight direction of Aquila.

**Figure 4:**
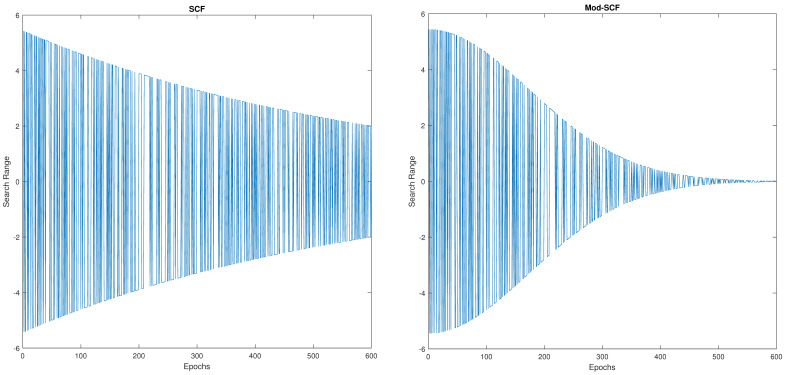
Comparison between IAO and MAO in reaching convergence.

A comparison between SCF and M-SCF in approaching convergence is shown in Figure 4. In order to enhance the search accuracy, our M-SCF function aims to achieve faster convergence by restricting Aquila’s movement. Moreover, it reduces the optimization latency. Compared to the original AO, our modified approach takes less time to identify the optimal solution set. Both optimization algorithms have been implemented with population sizes of 250 and 250 epochs. A comparison table is given below in Table 6.

**Figure 5:**
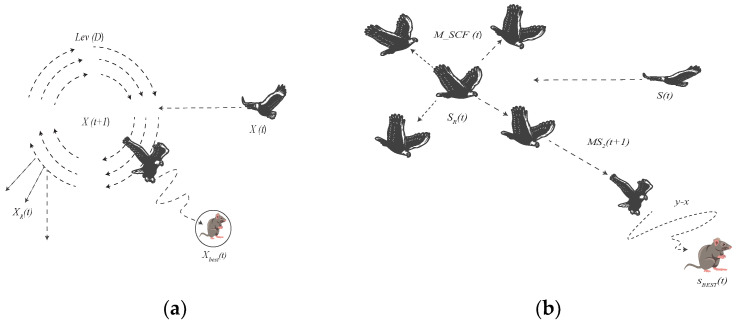
Full search with a short glide attack (**a**) Original AO, and (**b**) Modified AO.

**Figure 6:**
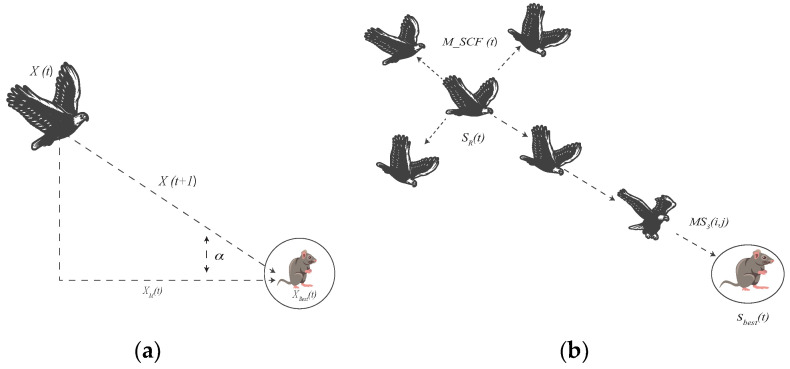
Search around prey and attack: (**a**) Original AO and (**b**) Modified AO.

Through the integration of the suggested M-SCF function, our proposed algorithm encompasses four distinct search steps, which can be described as follows:

1. Step 1: Vertical Dive Attack ($S_{1}$)
  Aquila starts its hunt by detecting the prey area and choosing the optimal hunting location by swooping high in the air. This attack is known as a vertical dive attack [41]. It can be represented as
  (11) $$S_{1}\left(t+1\right)=S_{best}\left(t\right)\times \left(1-\frac{t}{T}\right)+\left(S_{M}\left(t\right)-S_{best}\left(t\right)\times r\right)$$
  where $S_{1}\left(t+1\right)$ is the solution candidate of the (t+1) epoch, *r* is a random value between 0 and 1, and $S_{best}\left(t\right)$ is the best solution obtained up to the *i*-th generation. In the equation, $\left(1-\frac{t}{T}\right)$ is used to control the search space exploration process. Here, $S_{M}\left(t\right)$ is the mean value of the current solution to the *i*-th epoch.
2. Step 2: Modified Full Search with a Short Glide Attack ($MS_{2}$)
  Following the first searching step, Aquila thoroughly explores the target solution space using various directions and speeds before attacking the prey. This is known as a full search with a short glide attack [42], which is represented as
  (12) $$MS_{2}\left(t+1\right)=S_{R}\left(t\right)+M-SCF\left(t\right)\times \left(S_{best}\left(t\right)-S\left(t\right)\right)\times r\times \left(y-x\right)$$
  where x,y both correspond to the coordinates or positions of points forming the spiral shapes during the search process, *r* represents a random number between 0 and 1, and $M_{SCF}\left(t\right)$ is the modified search control factor which is defined in Equation (9). Instead of using the Levy Flight Distribution, we incorporated M-SCF to eradicate the problem of falling into local optima. Figure 5 portrays the difference compared to the original attack.
3. Step 3: Modified Search Around Prey and Attack ($MS_{3}$)
  After the $MS_{2}$ searching step, the prey’s area is precisely located. The Aquila searches around the prey thoroughly, and with a pseudo attack, identifies the prey’s reaction. This is known as Search Around Prey and Attack [42], which is represented as
  (13) $$MS_{3}\left(i,j\right)=lb_{j}+r\times \left(ub_{j}-lb_{j}\right)+r\times \left(S_{R}\left(j\right)-S_{best}\left(j\right)\right)\times M-SCF\left(t\right)\times \left(1-\frac{t}{T}\right)$$
  where $S_{R}\left(j\right)$ is a random solution set, and $MS_{3}\left(i,j\right)$ is the current solution for *t* epochs. Figure 6, illustrate the difference compared with the original attack.
4. Step 4: Walk and Grab Attack ($S_{4}$)
  Lastly, Aquila attacks from above, depending upon the prey’s motion for the fourth technique. This searching step is known as “Walk and Grab Prey” [41], which can be represented as
  (14) $$S_{4}\left(t+1\right)=Q_{F}\times S_{best}\left(t\right)-\left(G_{1}\times S\left(t\right)\times r\right)-G_{2}\times lev\left(D\right),$$
  (15) $$Q_{F}=t^{\frac{2\times r-1}{{\left(1-T\right)}^{2}}},$$
  (16) $$G_{1}=2\times random-1,$$
  (17) $$G_{2}=2\times \left(1-\frac{t}{T}\right).$$
  where $S_{4}\left(t+1\right)$ is denoted as the solution obtained with this technique, and lev(D) is the Levy Distribution for dimension space *D*. $Q_{F}$ is the quality function used to balance the search approach, $G_{1}$ indicates all kinds of movements of Aquila while hunting prey, and $G_{2}$ indicates the flight slope of Aquila’s hunt.

**Figure 7:**
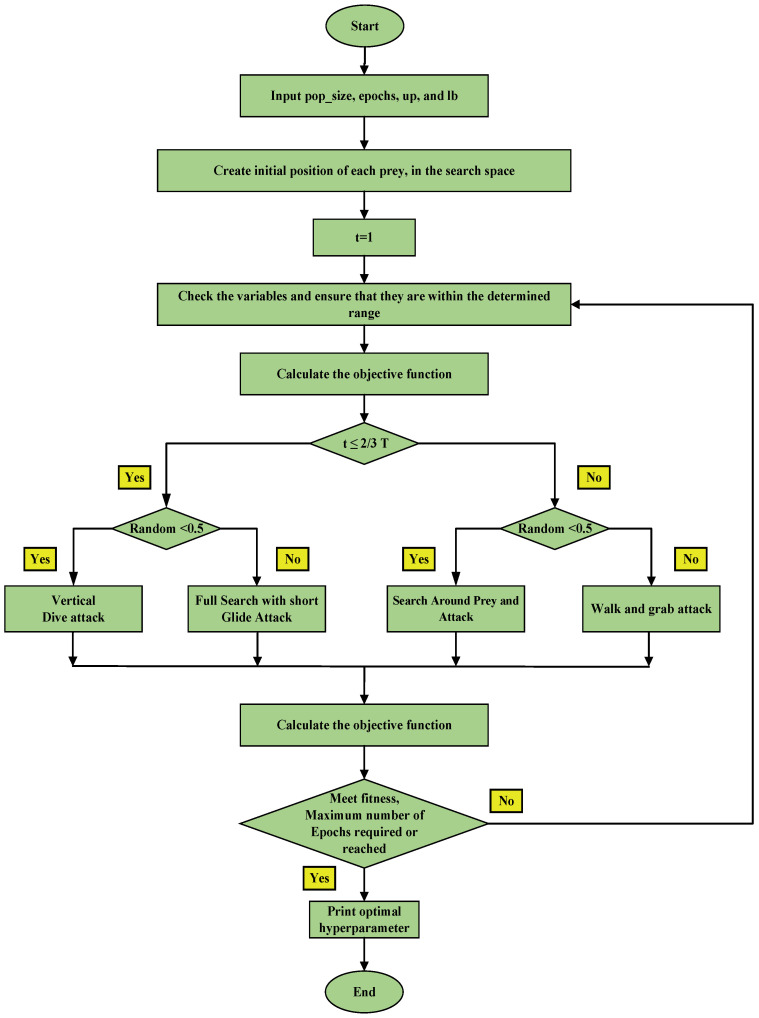
Flow chart of the Modified-AO.

The M-AO optimizer was specifically developed to solve the optimization problem associated with hyper-parameter tuning, as illustrated in the flowchart depicted in Figure 7.

## 4. Results

In this paper, we proposed an automatic model for neonatal seizure classification using EEG signals. After training several ML classifiers on the dataset acquired from Helsinki University Hospital, we compared the performances based on performance evaluation metrics. Additionally, we optimized XGBoost hyper-parameters with Original Aquila Optimization, which is termed AO-XGB. Along with this, we provided results based on a few other algorithms. This section depicts the performance chart, confusion matrix, and ROC curve for the mentioned classifiers. Finally, a comparison of the previously related research works was presented.

### 4.1. Confusion Matrix

**Figure 8:**
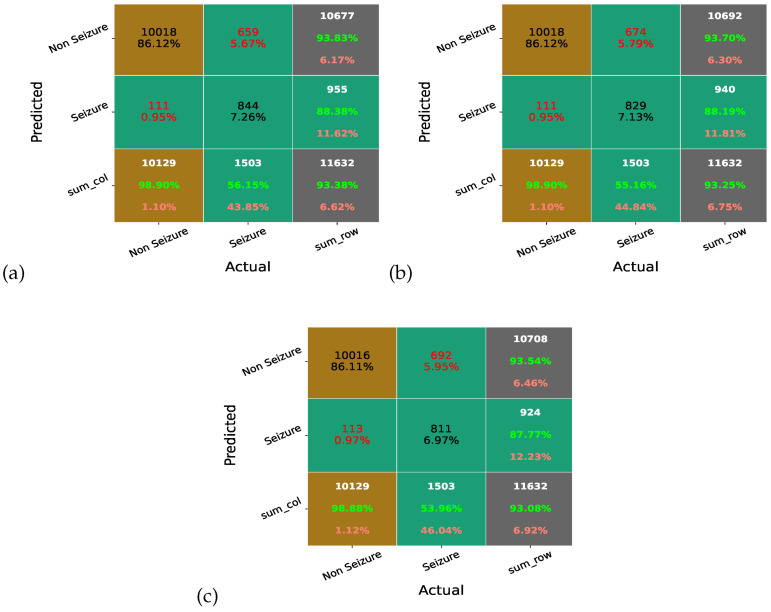
Confusion matrix of (**a**) MAO-XGB, (**b**) AO-XGB, and (**c**) Nor-XGB.

A classification problem’s prediction outcomes are compiled in a confusion matrix. Count values are used to describe the numbers of accurate and inaccurate predictions for each class. The confusion matrix is a measure of how well a classifier performs on any dataset. The off-diagonal elements reflect the number of times the classification model is misclassified, whereas the diagonal elements represent the actual or proper labels. As a result, the classification model improves and becomes more accurate with higher confusion matrix diagonal element values. Here, Figure 8 illustrates the confusion matrix of MAO-XGB, AO-XGB, and XGB without any optimization (Nor-XGB). We can see that the MAO-XGB detected 844 seizure cases, whereas the AO-XGB and Nor-XGB detected 829 and 811 cases, respectively. Thus, the MAO-XGB classifier is better and more accurate than AO-XGB and Nor-XGB.

**Figure 9:**
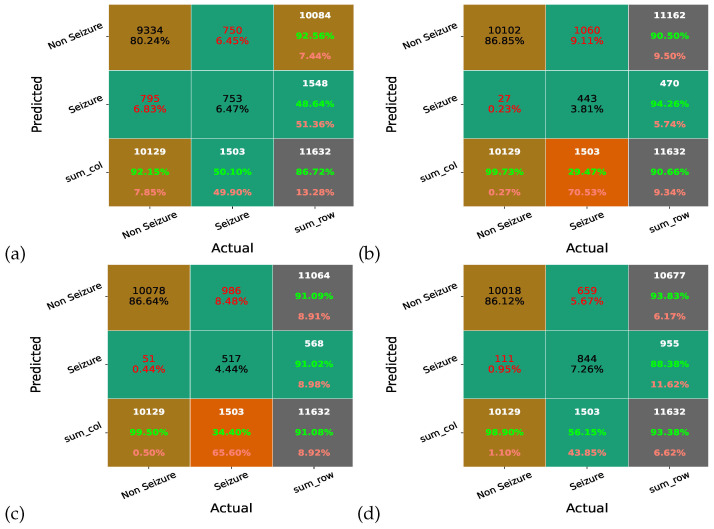
Confusion matrix of (**a**) DT, (**b**) RF, (**c**) GBC, and (**d**) MAO-XGB.

Moreover, Figure 9 illustrates the confusion matrix of MAO-XGB, RF, GBC, and DT. We can see that the RF, GBC, and DT detected 443, 753, and 517 seizure cases, respectively, significantly less than MAO-XGB. Thus, the MAO-XGB classifier is better and more accurate than the other trained classifiers.

### 4.2. Performance Chart

**Figure 10:**
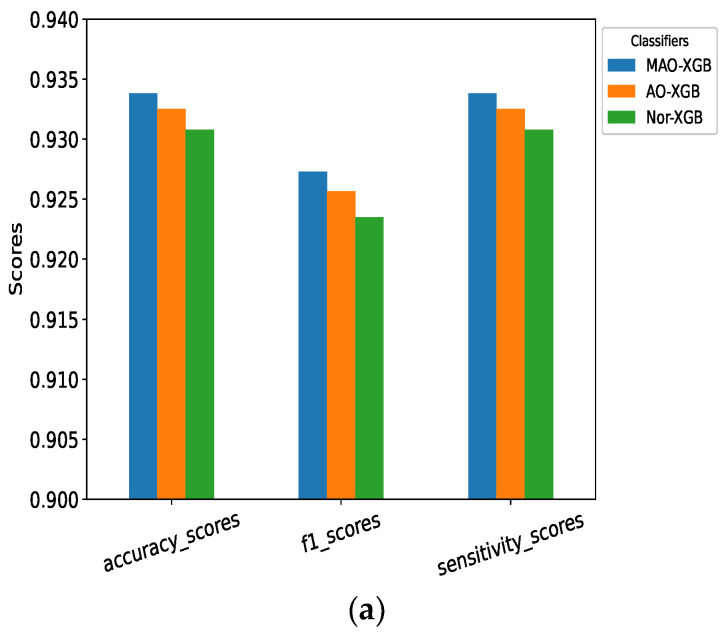

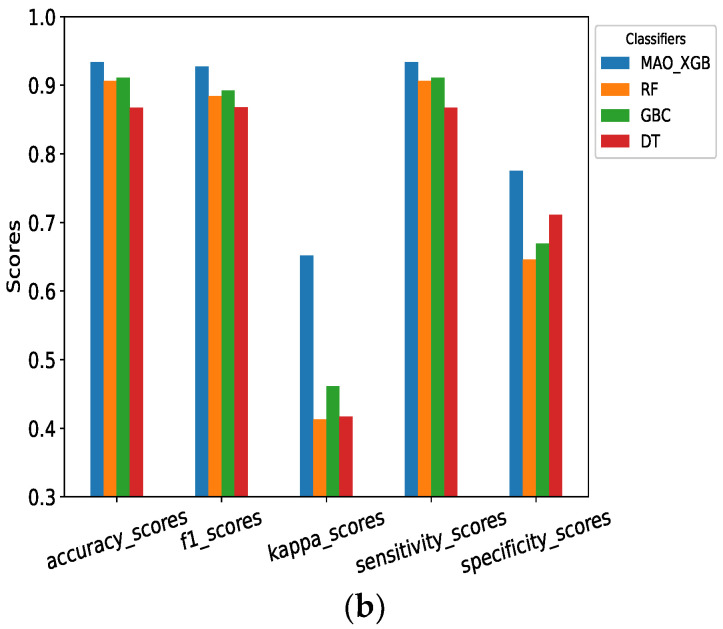
(**a**,**b**) Performance chart.

The performance chart compares the fruitfulness of different models. Figure 10 shows the performance chart of the MAO-XGB, AO-XGB, Nor-XGB, RF, DT, and GBC classifiers. We compared the accuracy score, F1 score, and sensitivity of MAO-XGB, AO-XGB, and Nor-XGB. To compare MAO-XGB with RF, GBC, and DT, we considered the accuracy score, F1 score, sensitivity, kappa, and specificity score.

From Figure 10 we can obtain a clear understanding that the performance of the MAO-XGB classifier outperforms the others, making it a better option for detecting neonatal seizures with higher accuracy and reliability.

### 4.3. ROC Curve

**Figure 11:**
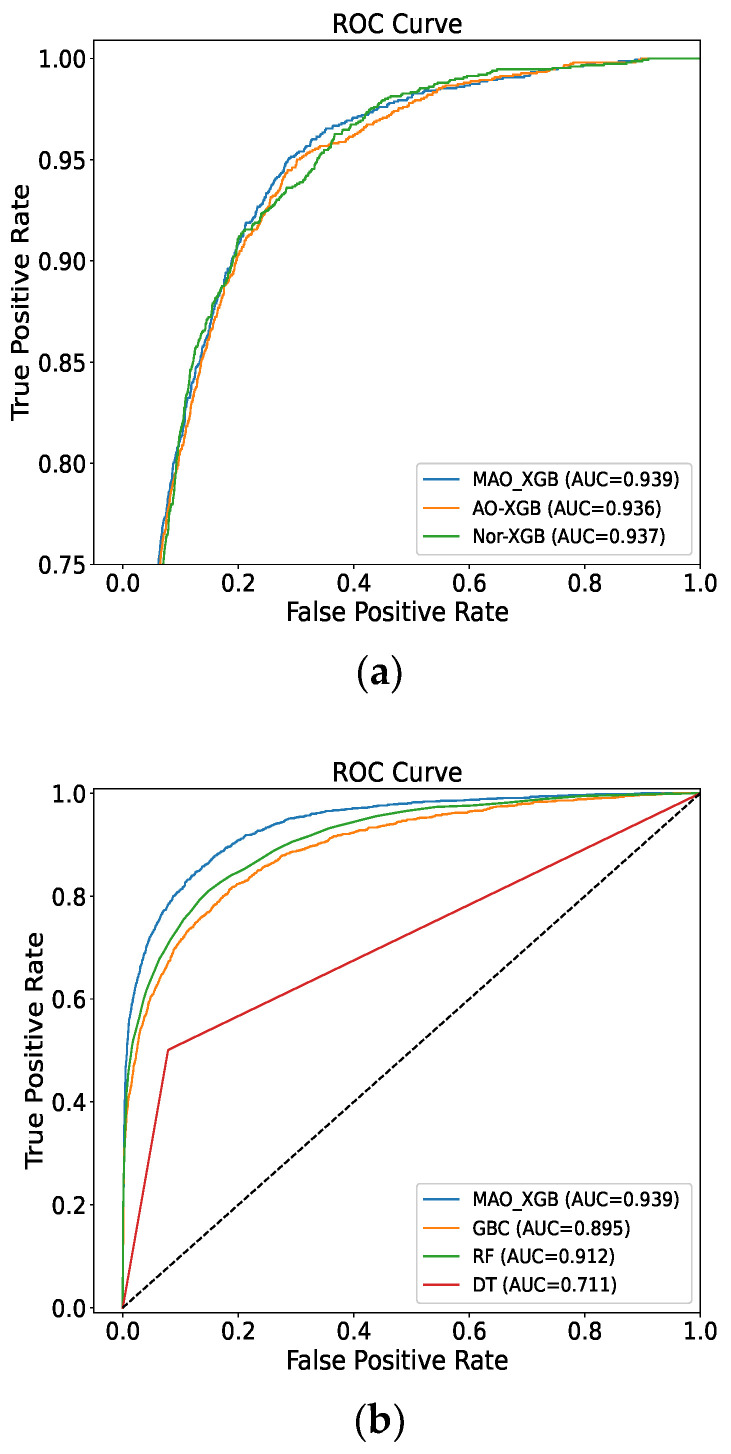
(**a**,**b**) ROC curve.

The ROC curve, also referred to as a receiver operating characteristic curve, graphically depicts the varying diagnostic performances of a binary classifier system by varying the discrimination thresholds. This curve is plotted based on the performance of the classifiers. This graph justifies that the MAO-XGB outperforms the other classifiers, as it covers a bigger area under the curve, as shown in Figure 11. Furthermore, the AUC value of MAO-XGB is 0.939, which is higher than that of other classifiers.

### 4.4. Comparison with Other Research

Although several research studies have included ML approaches for detecting neonatal seizures, our model showed a better performance than those described in most other studies. We compared our research with relevant studies that includes ML algorithms, for instance [9,10,11,12,13]. These are indicated with (✓). We did not consider the other studies, as they used adapted DL approaches, and these studies are indicated with (x). We observed that our model acquired an accuracy of 93.38%, a F1 score of 92.72%, a kappa score of 65.17%, a sensitivity of 93.38, and a specificity of 77.52%. For [9], we only considered the SVM classifier and did not compare the accuracy of deep learning classifiers such as ANN and CNN. Table 7 shows a summary of the related work, which indicates that our model acquired the best result compared to the other studies stated above.

## 5. Conclusions and Future Work

### 5.1. Conclusions

Seizures are a common neurological condition that frequently affects newborns and serves as a symptom of significant neurological disorders. Electroencephalography (EEG) data can reveal fluctuations in brain activity in neonates, aiding in the identification of brain disorders such as seizures. However, accurate interpretation requires the expertise of highly skilled medical personnel. To address this, machine learning (ML) algorithms can play a crucial role by offering time-efficient alternatives to traditional techniques. ML models can contribute significantly to neonatal seizure detection by providing more reliable results to distinguish between seizure and non-seizure states. Our proposed MAO-XGB model achieved an accuracy of 93.38%, which is higher than most of the available shallow learning models from relevant studies. Recent literature shows that a few variants of AO have been introduced to solve complex problems. Among these variants is IAO, which has demonstrated exceptional performances in certain cases. Inspired by this IAO, we proposed a modified version of AO, termed M-AO. This modification resulted in a smaller search space complexity. This M-AO was adapted to optimize the hyper-parameters of XGBoost to increase its reliability. We compared our optimized model with existing relevant research, which showed that our proposed model performs better and shows better accuracy. After completing our study, medical professionals will be able to easily and efficiently utilize this framework without requiring formal training, enabling them to quickly identify neonatal seizures. This paper marks the first instance of employing an M-AO-based optimized XGBoost model for neonate seizure detection.

### 5.2. Future Work

One of the main flaws in our research is that we could not gather any real-time data from actual seizure activity. The unbalanced dataset used in this investigation has a somewhat decreased level of accuracy. Additionally, collecting data from other countries such as Saudi Arabia or Bangladesh in real time would be revolutionary. We will also look into whether the preparation procedures lost any useful information. In the future, we will use better preprocessing techniques that incorporate all of the necessary information to recognize neonate seizures. With a highly balanced dataset, this model may work better and create a more robust system for neonatal seizure detection. In search of more accurate seizure detection, we will also apply DL algorithms such as LSTM and CNN on a balanced dataset in addition to ML algorithms. This may enable us to make our system more effective and increase the accuracy of the neonatal seizure detection process.

## Figures and Tables

**Table 1:** This table represents the incidence of neonatal seizures.

Area	Setting	Incidence	Ref.
USA	NICU (1992–1994)	Overall: 1.8/1000 live births	[2]
Canada	NICU (1990–1995)	Overall: 2.5/1000 live births	[3]
UK	NICU (2007–2008)	4%	[4]
India	NICU (2011–2013)	1.6%	[5]

**Table 2:** Description of the relevant papers.

No.	Algorithm	DL	ML	Remarks
[9]	SVM, ANN, 1D-CNN	Yes	Yes	The performance levels of the three models were compared, and CNN was proven to be the best among them with an accuracy level of 95.99%.
[10]	SVM	No	Yes	The developed SVM was used to detect the non-stationary periodic characteristics of neonatal seizures. But, a lack of hardware implementations reduced the clinical feasibility
[11]	ProtoNN	No	Yes	This architecture has a sensitivity of 87%, which is higher than related previous research. Because it can be optimized at 4.84 KB, the ML model used here can be deployed in wearable ultra-edge devices.
[12]	LR, dense tree, 2D SVM, Cos KNN	No	Yes	The relative time taken by the proposed ML algorithm was 62.5% of the baseline, which increased the performance of the algorithm and provided better accuracy for seizure detection. As the data dimensions were reduced by using the PCA, the performance of the training and test data improved.
[13]	SVM	No	Yes	The performance of the initial training set was cross-validated against the performance of the validation set to assess its generalizability. The accuracy of the proposed algorithm was 89–93%. Moreover, this study acquired an AUC of 99% and a Kappa of 68%.
[21]	GCNN	Yes	No	The findings from GCNN demonstrate that functional connectivity measures derived from EEG graph representations can effectively take advantage of the dependencies between EEG data and the results.
[15]	CNN, LSTM,	Yes	No	The algorithm correctly identified 71.6% of patients with seizures and 96.4% of normal patients as not having any seizures. The restrictions on improving the models’ accuracy levels were examined, and potential fixes were offered.
[16]	SWT	Yes	No	Appropriate time windows were selected for this program so that the non-stationarity of the signals and the artifacts did not affect the EEG recordings. The use of SWT increased the performance of the proposed methods by 5% compared to the process where a raw EEG time series was used.
[17]	ADA (Artefact Detection Algorithm)	Yes	No	When separating periods of clean, artifact-free EEG from any form of artifact, the Residual Deep Neural Network demonstrated great accuracy (95%), with a median accuracy of 91% for each patient. The five various forms of artifacts were correctly identified with accuracy levels of 57% to 92%.
[18]	2D- CNN	Yes	No	To avoid model overfitting, weighted loss parameters for the loss function were used in this study. As a result, data upscaling and downscaling, as well as information loss, can be avoided to a large extent. This study showed increased ACC and AUC results, where small time windows of 1 s were used for evaluation.
[19]	CNN	Yes	No	This research converted the EEG signals into color images and used them as inputs for the pre-trained DCNN. The transfer learning framework used here helped to eliminate the hyper-parameter optimization phase more efficiently compared to other deep neural networks.
[20]	CNN	Yes	No	A sliding window design was developed for the training data generation process. This design increased the amount of data available to feed into neural networks on a large scale and this data augmentation worked effectively for the research. It helped the researchers to analyze their dataset by performing some modifications in the R and Python codes.
Proposed Model	MAO-XGB	No	Yes	Our paper aimed to develop an ML framework for better seizure detection, optimize the AO algorithm for faster convergence, and fine-tune hyper-parameters using a modified MHOA approach to improve the overall performance.

**Table 3:** Feature Descriptions.

Name	Type	Equation
Mean	Time Domain Features	$M=\frac{sum\;of\;the\;signal\;values}{total\;number\;of\;signal\;values}$
Median		$MV=(\frac{n+1}{2})th\;value\;of\;thesignalset$
Variance		V=X−Y
Rms		$X_{rms}=\sqrt{\frac{\left({\left(x_{1}\right)}^{2}+{\left(x_{2}\right)}^{2}+\ldots .+{\left(x_{n}\right)}^{2}\right)}{n}}$
Standard Deviation		$\sigma \;=\sqrt{\frac{\sum ((|X_{1}-{µ|)}^{2})}{N}}$
Skewness		$S=\;3\times \;\frac{mean-median}{standard\;deviation}$
Kurtosis		$K=\;n\times \frac{\sum_{i}^{n}{\left(Y_{i}-µ\right)}^{4}(\sum_{i}^{n}{\left(Y_{i}-µ\right)}^{2})2}{N}$
Interquartile Range		$The\;interquartile\;range,\;I=\;Q_{3}-Q_{1}$
Hjorth Activity		HA=var(x(t))
Hjorth Mobility		$HM=\;\sqrt{\frac{var(\frac{dx\left(t\right)}{dt})}{var(x\left(t\right))}}$
Hjorth complexity		$HC=\;\frac{Mobility\;(\frac{dx\left(t\right)}{dt})}{Mobility\;(x\left(t\right))}$
Permutation Entropy	Entropy Domain Features	$PE=\;-\;\sum_{m=1}^{m!}p\left(\pi \right)lnln\;p\left(\pi \right)$
Shannon Entropy		$H\left(x\right)=\;-\;\sum_{i=1}^{n}p\left(x_{i}\right)loglog\;p\left(\;x\right)$
Approximate Entropy		ApEn(m,r,N)(u)=ømr−øm+1(r)
Sample Entropy		$S=\;-\;ln\;\frac{A}{B}$

**Table 4:** Performance Comparisons of Tree-Based Algorithms.

Classifier	ACC	Re	F1	K
XGBoost	92.80%	53.89%	65.93%	62.13%
RF	90.62%	28.8%	44.25%	40.69%
GBC	90.86%	33.39%	45.43%	44.67%
DT	85.52%	46.64%	48.57%	37.09%

**Table 5:** Hyper-parameters of the XGBoost classifier.

Hyper-Parameters	No. of Hyper-Parameters	Lower Limit	Upper Limit
Learning Rate	7	1.5 × 10${}^{-15}$	0.9
Colsample by tree		0.001	1.00
Gamma		1 × 10${}^{-9}$	1.0
Max Depth		1	200
Subsample		1	200
Min Child Weight		1	200
Alpha		1 × 10${}^{-6}$	1.0

**Table 6:** Run Time Comparison between AO and M-AO.

Algorithms	Time (Hours)
Original Aquila Optimization	51.27
Modified Aquila Optimization	41.05

**Table 7:** Comparison with related work.

Model Type	Work	Algorithms	Accuracy	GDR	AUC	Sensitivity	Specificity	AUC	Kappa Score	F1 Score	Median AUC	Median AUC90	Remarks
Machine Learning	[9]	SVM	92.30%	-	-	-	-	-	-	-	-	-	✓
	[10]	SVM	-	-	98%	-	-	-	-	-	-	-	✓
	[11]	ProtoNN	-	-	-	87%	-	-	-	-	-	-	✓
	[12]	LR	56.48%	-	-	-	-	-	-	-	-	-	✓
		DT	45.48%	-	-	-	-	-	-	-	-	-	✓
		2D-SVM	68.14%	-	-	-	-	-	-	-	-	-	✓
		Cos-KNN	68.14%	-	-	-	-	-	-	-	-	-	✓
	[13]	SVM	89–93%		99%	-	-	-	68%	-	-	-	✓
Deep Learning	[9]	CNN	99%	-	-	-	-	-	-	-	-	-	{x}
		ANN	88%	-	-	-	-	-	-	-	-	-	{x}
	[21]	GCNN	-	-	-	-	-	-	-	-	99.1%	96%	{x}
			-	-	-	-	-	-	-	-	99%	95.7%	{x}
			-	-	-	-	-	-	-	-	97.3%	94.9%	{x}
	[15]	CNN	74.3%	-	-	-	-	-	-	-	-	-	{x}
		LSTM	74.3%	-	-	-	-	-	-	-	-	-	{x}
	[16]	SWT	-	77%	81%	-	-	-	-	-	-	-	{x}
	[17]	ADA	95%	-	97%	-	-	-	-	-	-	-	{x}
	[18]	2D-CNN	95.6%	-	-	-	-	-	-	-	-	-	{x}
			94.8%	-	-	-	-	-	-	-	-	-	{x}
			90.1%	-	-	-	-	-	-	-	-	-	{x}
	[19]	CNN	91.38%	-	-	-	-	-	-	-	-	-	{x}
		Alexnet	95.96%	-	-	-	-	-	-	-	-	-	{x}
		Resnet18	97.45%	-	-	-	-	-	-	-	-	-	{x}
		Google net	94.42%	-	-	-	-	-	-	-	-	-	{x}
		Dense net	97.93%	-	-	-	-	-	-	-	-	-	{x}
	[20]	CNN	96–97%	-	-	-	-	-	-	-	-	-	{x}
Proposed Model		**MAO-XGB**	**93.38%**	-	-	**93.38%**	**77.52%**	-	-	**92.72%**	-	-	

## Data Availability

The processed data, trained model, and codes related to this study are available at: https://github.com/MIrazul29/NSD_MAO-XGB.git. The original datset is available at: https://zenodo.org/record/1280684 and https://www.nature.com/articles/sdata201939#Sec7.

## References

[1] Greene B.R., Faul S., Marnane W.P., Lightbody G., Korotchikova I., Boylan G.B. (2008). A comparison of quantitative EEG features for neonatal seizure detection. Clin. Neurophysiol..

[2] Saliba R.M., Annegers J.F., Waller D.K., Tyson J.E., Mizrahi E.M. (1999). Incidence of neonatal seizures in Harris County, Texas, 1992–1994. Am. J. Epidemiol..

[3] Ronen G.M., Penney S., Andrews W. (1999). The epidemiology of clinical neonatal seizures in Newfoundland: A population-based study. J. Pediatr..

[4] Shah D.K., Zempel J., Barton T., Lukas K., Inder T.E. (2010). Electrographic seizures in preterm infants during the first week of life are associated with cerebral injury. Pediatr. Res..

[5] Ghanshyambhai P., Sharma D., Patel A., Shastri S. (2016). To study the incidence, etiology and EEG profile of neonatal seizures: A prospective observational study from India. J. Matern.-Fetal Neonatal Med..

[6] Tekgul H., Gauvreau K., Soul J., Murphy L., Robertson R., Stewart J., Volpe J., Bourgeois B., du Plessis A.J. (2006). The current etiologic profile and neurodevelopmental outcome of seizures in term newborn infants. Pediatrics.

[7] Costea R.M., Maniu I., Dobrota L., Pérez-Elvira R., Agudo M., Oltra-Cucarella J., Dragomir A., Bacilă C., Banciu A., Banciu D.D. (2021). Exploring Inflammatory Status in Febrile Seizures Associated with Urinary Tract Infections: A Two-Step Cluster Approach. Brain Sci..

[8] Pisani F., Orsini M., Braibanti S., Copioli C., Sisti L., Turco E.C. (2009). Development of epilepsy in newborns with moderate hypoxic-ischemic encephalopathy and neonatal seizures. Brain Dev..

[9] Elakkiya R. (2021). Machine learning based intelligent automated neonatal epileptic seizure detection. J. Intell. Fuzzy Syst..

[10] Tapani K., Vanhatalo S., Stevenson N.J. (2019). Time-varying EEG correlations improve automated neonatal seizure detection. Int. J. Neural Syst..

[11] Nagarajan V., Muralidharan A., Sriraman D., Kumar P. Scalable Machine Learning Architecture for Neonatal Seizure Detection on Ultra-Edge Devices. Proceedings of the 2022 2nd International Conference on Artificial Intelligence and Signal Processing (AISP).

[12] Ryu S., Back S., Lee S., Seo H., Park C., Lee K., Kim D.S. (2021). Pilot study of a single-channel EEG seizure detection algorithm using machine learning. Child’s Nerv. Syst..

[13] Tapani K., Nevalainen P., Vanhatalo S., Stevenson N.J. (2022). Validating an SVM-based neonatal seizure detection algorithm for generalizability, non-inferiority and clinical efficacy. Comput. Biol. Med..

[14] Raeisi K., Khazaei M., Croce P., Tamburro G., Comani S., Zappasodi F. (2022). A graph convolutional neural network for the automated detection of seizures in the neonatal EEG. Comput. Methods Programs Biomed..

[15] Zeedan A., Al-Fakhroo K., Barakeh A. EEG-Based Seizure Detection Using Feed-Forward and LSTM Neural Networks Based on a Neonates Dataset. Proceedings of the 4th International Conference on Applied Engineering and Natural Sciences.

[16] Frassineti L., Ermini D., Manfredi C., Fabbri R. Neonatal seizures detection using stationary wavelet transform and deep neural networks: Preliminary results. Proceedings of the 2020 IEEE 20th Mediterranean Electrotechnical Conference (MELECON).

[17] Webb L., Kauppila M., Roberts J., Vanhatalo S., Stevenson N. (2021). Automated detection of artefacts in neonatal EEG with residual neural networks. Comput. Methods Programs Biomed..

[18] Tanveer M., Khan M., Sajid H., Naseer N. (2021). Convolutional neural networks ensemble model for neonatal seizure detection. J. Neurosci. Methods.

[19] Caliskan A., Rencuzogullari S. (2021). Transfer learning to detect neonatal seizure from electroencephalography signals. Neural Comput. Appl..

[20] Gramacki A., Gramacki J. (2022). A deep learning framework for epileptic seizure detection based on neonatal EEG signals. Sci. Rep..

[21] Li Z., Wang Q., Zhu B., Wang B., Zhu W., Dai Y. (2022). Thermal error modeling of high-speed electric spindle based on Aquila Optimizer optimized least squares support vector machine. Case Stud. Therm. Eng..

[22] Stevenson N., Tapani K., Lauronen L., Vanhatalo S. (2019). A dataset of neonatal EEG recordings with seizure annotations. Sci. Data.

[23] Mathieson S., Livingstone V., Low E., Pressler R., Rennie J.M., Boylan G.B. (2016). Phenobarbital reduces EEG amplitude and propagation of neonatal seizures but does not alter performance of automated seizure detection. Clin. Neurophysiol..

[24] Isaev D., Tchapyjnikov D., Cotten C.M., Tanaka D., Martinez N., Bertran M., Sapiro G., Carlson D. (2020). Attention-based network for weak labels in neonatal seizure detection. Proc. Mach. Learn. Res..

[25] Pitfalls of Filtering the EEG Signal—Sapien Labs. Neuroscienc. Human Brain Diversity Project. https://sapienlabs.org/lab-talk/pitfalls-of-filtering-the-eeg-signal/.

[26] Al-Fahoum A.S., Al-Fraihat A.A., Grant A., Hinojosa J.A., Oliveira M.S. (2014). Methods of EEG signal features extraction using linear analysis in frequency and time-frequency domains. Int. Sch. Res. Not..

[27] Übeyli E.D. (2009). Statistics over features: EEG signals analysis. Comput. Biol. Med..

[28] IBM What is a Decision Tree. https://www.ibm.com/topics/decision-trees.

[29] Decision Tree—Overview, Decision Types, Applications. https://corporatefinanceinstitute.com/resources/data-science/decision-tree/#:~:text=Decision%20trees%20are%20used%20for,and%20continuous%20variable%20decision%20trees.

[30] Wang X., Gong G., Li N., Qiu S. (2019). Detection analysis of epileptic EEG using a novel random forest model combined with grid search optimization. Front. Hum. Neurosci..

[31] What is Gradient Boosting in Machine Learning? Boosting Algorithm. https://intellipaat.com/blog/gradient-boosting-in-machine-learning/?US.

[32] Gradient Boosting—Overview, Tree Sizes, Regularization. https://corporatefinanceinstitute.com/resources/data-science/gradient-boosting/.

[33] XGBoost—What Is It and Why Does It Matter?. https://www.nvidia.com/en-us/glossary/data-science/xgboost/.

[34] Machine Learning with XGBoost and Scikit-learn. https://www.section.io/engineering-education/machine-learning-with-xgboost-and-scikit-learn/.

[35] Balli O. Use of XGBoost Algorithm in Classification of EEG Signals. Proceedings of the 1st International Conference on Engineering, Natural and Social Sciences.

[36] Wang F., Tian Y.C., Zhang X., Hu F. (2022). An ensemble of Xgboost models for detecting disorders of consciousness in brain injuries through EEG connectivity. Expert Syst. Appl..

[37] Yu Y., Qiu W., Quan C., Qian K., Wang Z., Ma Y., Hu B., Schuller B.W., Yamamoto Y. Federated Intelligent Terminals Facilitate Stuttering Monitoring. Proceedings of the ICASSP 2023—2023 IEEE International Conference on Acoustics, Speech and Signal Processing (ICASSP).

[38] Dhaliwal S.S., Nahid A.A., Abbas R. (2018). Effective intrusion detection system using XGBoost. Information.

[39] Awal M.A., Masud M.S., Hossain M.S., Bulbul A.A.-M., Mahmud S.M.H., Bairagi A.K. (2021). A novel bayesian optimization-based machine learning framework for COVID-19 detection from inpatient facility data. IEEE Access.

[40] Mirjalili S. (2019). Evolutionary Algorithms and Neural Networks.

[41] Abualigah L., Yousri D., Elaziz M.A., Ewees A.A., Al-Qaness M.A., Gandomi A.H. (2021). Aquila optimizer: A novel meta-heuristic optimization algorithm. Comput. Ind. Eng..

[42] Gao B., Shi Y., Xu F., Xu X. (2022). An improved Aquila optimizer based on search control factor and mutations. Processes.

